# The role of GAGE cancer/testis antigen in metastasis: the jury is still out

**DOI:** 10.1186/s12885-015-1998-y

**Published:** 2016-01-08

**Authors:** Morten Frier Gjerstorff, Mikkel Green Terp, Malene Bredahl Hansen, Henrik Jørn Ditzel

**Affiliations:** Department of Cancer and Inflammation Research, Institute for Molecular Medicine, University of Southern Denmark, Winsloewparken 25, 3, Odense, DK-5000 Denmark; Department of Oncology, Odense University Hospital, Sdr. Boulevard 29, Odense, DK-5000 Denmark

## Abstract

**Background:**

GAGE cancer/testis antigens are frequently expressed in various types of malignancies and represent attractive targets for immunotherapy, however their role in cancer initiation and progression has remained elusive. GAGE proteins are expressed in normal cells during early development with migratory and invasive properties and were found to be upregulated in cancer cells with metastasizing potential in a gastric cancer model.

**Methods:**

We have addressed the direct role of GAGE proteins in supporting metastasis using an isogenic metastasis model of human cancer, consisting of 4 isogenic cell lines, which are equally tumorigenic in immunodeficient mice, but differ with their ability to generate metastases in the lungs and lymph nodes.

**Results:**

Although GAGE proteins were strongly upregulated in the highly metastatic clone (CL16) compared to non-metastatic (NM2C5), weakly metastatic (M4A4) and moderately metastatic clones (LM3), stable downregulation of GAGE expression did not affect the ability of CL16 cells to establish primary tumors and form metastasis in the lungs of immunodeficient mice.

**Conclusions:**

These results suggest that GAGE proteins *per se* do not support metastasis and that further studies are needed to clarify the contribution of GAGE proteins to the metastatic potential of different types of cancer cells.

## Background

Metastasis is the cause of most cancer-related deaths and remains the most significant challenge to management of the disease. Thus it is essential to gain more insight into the mechanisms of the metastatic process. Several lines of evidence link the GAGE cancer/testis antigen family to cancer metastasis. This is an highly interesting observation since GAGE proteins have attracted significant interest as potential targets for immunotherapy due to their near cancer-specific expression and ability to elicit immune responses in patients [1]. During early human development, GAGE proteins are expressed in the cells of the trophectoderm [2], which invade the uterine tissue during blastocyst implantation, and in primordial germ cells when they migrate from the yolk sac to colonize the fetal testis [3]. Furthermore, knockdown of GAGE proteins in melanoma cell lines has been demonstrated to significantly reduce their ability to migrate [4]. Thus, there is clearly a potential link between GAGE proteins and a migratory and invasive phenotype that deserves further investigation.

In a recent study Lee et al. reported on a metastasis model of gastric cancer and identified a panel of genes differentially expressed in primary tumors vs. corresponding distant metastasis using mRNA microarray [5], including members of the GAGE12 family. Based on further experiments, the authors concluded that GAGE12 mediates human gastric carcinoma metastasis, but we find no direct data presented to supports this. Knockdown of GAGE12 expression in cancer cells derived from metastasis was shown to reduce the ability of the cells to form primary tumors when injected into the gastric wall of immunodeficient mice, but the ability of the cells to form metastasis at distant sites was not investigated. The relationship between primary tumor size, tumor cell dissemination and metastasis are complicated. For instance, it has been shown that some tumors disseminate metastasizing cells at an early stage and that these cells remain dormant at ectopic sites and subsequently undergo somatic progression, inducing metastatic growth. Thus, the size of the primary tumor cannot be regarded as a direct measure of metastasis development [6–8] and the study by Lee et al. did not demonstrate a causal role for GAGE12 in metastasis.

We have also been intrigued by whether GAGE proteins play a role in tumor metastasis and have investigated this in an isogenic breast cancer metastasis model.

## Methods

### Cell culture

All cell lines were culture in DMEM, supplemented with 10 % FBS (Invitrogen), penicillin (100 U/ml) and streptomycin (100 mg/ml) and kept at low passage for no more than 3 months. The identity of the cell lines was confirmed using the Cell ID System (Promega, Madison, WI, USA).

### Lentiviral packaging of shRNA plasmids

HEK293T cells were transfected with packaging plasmids pMD2.g, pRSV-Rev and pMDL g/p RRE (kindly provided by Didier Trone through Addgene, Cambridge, MA, USA) and shRNA plasmids targeting homologous regions of all known GAGE family members (purchased from Sigma Aldrich, Brondby, Denmark;) or control plasmid pLKO1. Target sequences for shRNAs were: GAGE-shRNA1 (TRCN0000137608), 5’- CCA AAT CCA GAG GAG GTG AAA -3’; GAGE-shRNA2 (TRCN0000137973), 5’- AGT GTG AAG ATG GTC CTG AT -3’; GAGE-shRNA3 (TRCN0000138463), 5’- CTC CTG AAA TGA TTG GGC CTA C -3’; GAGE-shRNA4 (TRCN0000136873), 5’- CAG TTC AGT GAT GAA GTG GAA -3’; GAGE-shRNA5 (TRCN0000137684), 5’- GAA CCA GCA ACA CCT GAA GAA -3’. After 72 h, lentivirus-containing media was harvested and stored at -80 degrees.

### Lentiviral transductions

Cells were seeded at a density of 20,000 cells/cm^2^ and the next day transduced in media with 5 μg/ml of polybrene. After 16 h media was changed and after another 48 h 0.2 μg/ml of puromycin was added to select stable transfectants. Cells were used for experiments after two passages in selective media.

### Western blotting

Sub-confluent monolayers of cells were washed twice in PBS, lysed in RIPA buffer for 30 min on ice and cleared by centrifugation at 15.000 rpm for 10 min at 4 °C. Samples were resolved by 4–20 % SDS-PAGE and electroblotted onto a PVDF membrane. The membrane was incubated in PBS, 0.1 % Tween-20, and 5 % non-fat dry milk powder to block remaining protein binding sites, and then incubated with anti-GAGE mAb M3 (1/5000) [9] followed by horseradish peroxidase conjugated goat anti-mouse IgG (1/100.000) (DakoCytomation Denmark A/S, Glostrup, Denmark). All antibody incubations and washing steps were carried out in PBS, 0.1 % Tween-20 and 1 % non-fat dry milk powder. The immunoreactive bands were visualized with ECL Western Blot kit (Amersham Biosciences, Hilleroed, Denmark).

### Immunohistochemical analysis

Cultured cells for immunostaining were fixed in 4 % formaldehyde for 24 h, prepared as cellblocks using Shandon Cytoblock (Thermo Electron Corporation, Pittsburg, PA, USA) and embedded in paraffin. Tissue sections were cut, deparaffinized, treated with 1.5 % H_2_0_2_ in Tris-buffered saline (pH 7.5) for 10 min to block endogenous peroxidase activity, rinsed in distilled H_2_O, demasked for antigen retrieval and washed in TNT buffer (0.1 M Tris, 0.15 M NaCl, 0.05 % Tween-20, pH 7.5). Anti-GAGE (clone M3) [9] was diluted 1:100 in antibody diluent (DAKO Cytomation, Glostrup, Denmark) and added to sections for 1 h at room temperature. Sections were washed with TNT and incubated with horseradish peroxidase-conjugated Envision (DAKO Cytomation) for 30 min, followed by another wash with TNT. The final reaction product was visualized by incubating with 3,3’-diaminobenzidine (DAB) + substrate-chromogen for 10 min, followed by washing with H_2_O and counterstaining of sections with Mayers hematoxylin before mounting in AquaTex (Merck Inc., Whitehouse Station, NJ, USA).

### Xenograft metastasis model

Evaluation of the effect of GAGE on the formation of primary tumors and spontaneous metastasis was carried out as described previously [10]. This study and its protocols were approved by the Animal Experiments Inspectorate (ID: 2014–15–0201–00128).

## Results and discussion

**Fig. 1:**
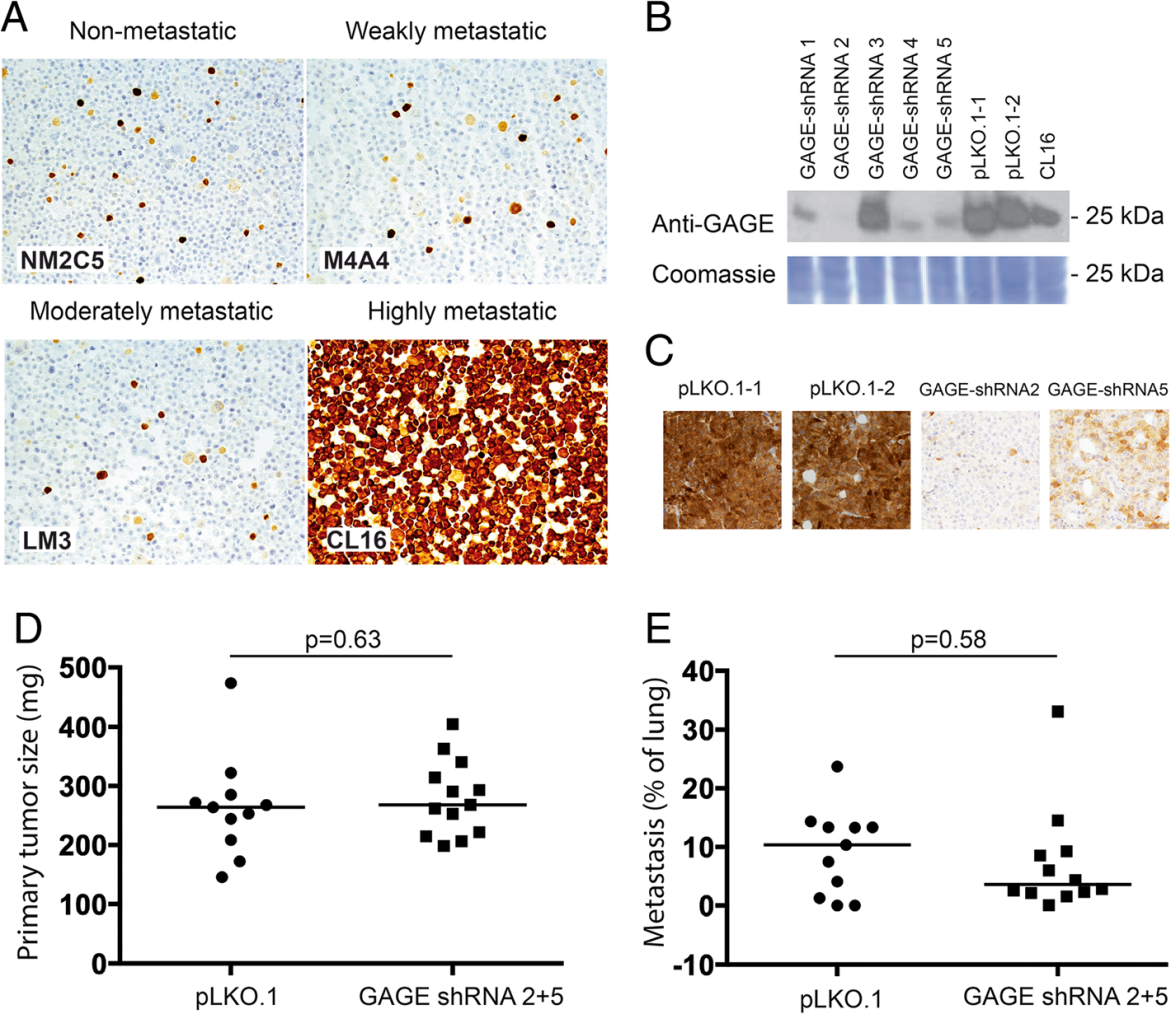
Characterization of the effect of GAGE proteins on the metastatic potential of breast cancer cells. **a** Immunohistochemical staining of GAGE proteins in MDA-MB-435-derived cell lines with different metastatic potential as described in Montel et al. [11] (anti-GAGE mAb, clone M4 [9]; DAB). **b** Western blot analysis of GAGE in CL16 cancer cells transduced with 5 different GAGE-specific lentiviral shRNA vectors (GAGE-shRNA1–5), empty vector (pLKO.1-1 and pLKO.1–2) or untransduced (CL16). **c**-**d** GAGE expression (C) and size (D) of primary tumors from CB17 mice implanted with 10^6^ GAGE-shRNA or pLKO.1-transduced CL16 cells and Matrigel (Sigma-Aldrich, St. Luis, Missouri, USA) into the mammary fat pat. **e** Quantification of the metastasis burden in lungs of mice by staining of the excised and embedded lungs with an antibody specific to human vimentin and scoring using NDP view software. Experimental groups were compared using the Students *t*-test (*p* values >0.05 was considered nonsignificant)

We have addressed the metastatic potential of GAGE proteins using an isogenic breast cancer metastasis model, derived from the metastatic breast carcinoma cell line MDA-MB-435 [11]. Interestingly, we found that the highly metastatic clone [M4A4-LM3-4 CL16 (CL16)] was highly enriched with GAGE-positive cells compared to non-metastatic (NM2C5), weakly metastatic (M4A4) and moderately metastatic clones [M4A4-LM3–2 (LM3)] (Fig. 1a). NM2C5, M4A4 and LM3 all had less than 1 % positive cell, similar to the original MDA-MB-431 cell line, while all cells of the CL16 clone were positive. NM2C5 and M4A4 are equally tumorigenic in immunodeficient mice, but only the latter produce metastases in the lungs and lymph nodes. Although NM-2C5-derived primary tumors disseminate single cells to the lungs, they remain dormant and do not form metastases. The moderately metastatic LM3 and highly metastatic CL16 cell lines were raised by cyclically culturing and orthotopically re-inoculating the cells of successive generations of metastases [11]. To examine whether GAGE proteins were directly implicated in the increased metastatic potential of CL16, we knocked down GAGE expression in these cells using stable lentiviral transductions with shRNA vectors (Fig. 1b) and investigated changes in the ability to metastasize. CL16 cells with shRNA-mediated knockdown of all known GAGE members (GAGE-shRNA2 and GAGE-shRNA5) and vector-only controls (pLKO1.1-1 and pLKO.1–2) were orthotopically transplanted into the mammary fat pads of female CB17 SCID mice. Five weeks later the primary tumors were surgically removed, having reached a size of 1.2 cm and knock down of GAGE expression was confirmed by immunohistochemistry (Fig. 1c and d). After another two weeks, the mice were sacrificed and the lungs removed for analysis of the metastatic burden. Tumor cells were identified in lung sections by staining of human vimentin, and the total size of tumors was quantified relative to lung size (Fig. 1e). This analysis demonstrated no significant difference in metastatic burden between the GAGE knockdown and control groups. Thus, GAGE proteins do not seem essential for the metastatic capability of CL16 breast cancer cells. Because GAGE proteins may have significantly diverse functions in different cancer types, GAGE expression should be considered in the context of the essential signaling pathways in the respective cancer cells. Thus, it is possible that GAGE proteins mediate metastasis in other experimental models.

## Conclusions

Based on currently available data, it cannot be concluded that GAGE proteins play a role in metastasis. Tumor antigens with direct roles in cancer development and progression are considered prime targets for immunotherapy, and thus the function of GAGE proteins in cancer cells should be further characterized. The gastric cancer metastasis model reported by Lee et al. may provide the basis for future studies directly addressing the involvement of GAGE proteins in the metastatic process.
